# Editorial: Working Dogs: Form and Function, Volume II

**DOI:** 10.3389/fvets.2021.732304

**Published:** 2021-07-29

**Authors:** Nathaniel J. Hall, Cynthia M. Otto, Wendy I. Baltzer

**Affiliations:** ^1^Department of Animal and Food Science, Texas Tech University, Lubbock, TX, United States; ^2^Penn Vet Working Dog Center, Philadelphia, PA, United States; ^3^Department of Clinical Sciences and Advanced Medicine, School of Veterinary Medicine, University of Pennsylvania, Philadelphia, PA, United States; ^4^Faculty of Science, The University of Sydney, Sydney, NSW, Australia

**Keywords:** olfaction, stress, behavior, service dog, detection dog, musculoskeletal, human animal bond, occupational hazards

## Introduction

Working dogs span the spectrum of careers from high powered physical athletes working in protection fields to highly cognitive dogs working in the service sector. Regardless of the career, all working dogs share a common requirement for physical capabilities and mental aptitude and partnership with their handler. In this Research Topic, Working Dogs: Form and Function, twenty manuscripts address the development and assessment of the physical Form, behavioral selection of dogs that contribute to Function, a focus on olfactory Function, occupational hazards that interfere with Form and Function and the relationship between the working dog and handler that addresses Form and Function of the working dog team.

## Occupational Hazards/Stressors

Working dogs perform tasks in a variety of domains. Dogs must adapt both physically and mentally to work regardless of the environment. Physical stamina is necessary for many working dogs. During exercise, these dogs must maintain physiologic homeostasis and mental acuity for prolonged periods of time. The most common physical stresses that impact performance are environmental temperatures, particularly heat stress and the physical wear and tear of high impact activity. Hydration is a critical factor in maintaining function and reducing the risk of heat injury (1). In a study of tracking dogs in the desert, the median working body temperature was 41C (106F) (Niedermeyer et al.). Dogs receiving electrolyte solutions or flavored water had increased fluid consumption compared to dogs receiving plain water. No adverse effects of electrolyte solutions were observed, however, chicken-flavored water without electrolytes was associated with increased markers of muscle injury.

Heat from muscle activity or the environment can lead to physiological stress and cellular injury. The gastrointestinal tract is affected by both physical stress (exercise, heat) (2) and mental stress (3). Many working dogs have diarrhea during or after intense exercise, which could be influenced by diet or physical conditioning. A group of nine hunting dogs in Italy were monitored over time to determine the effect of training, hunting and off-season rest on fecal stress markers and the microbiota (Zannoni et al.). Training and hunting did not detectably alter most of the stress markers, and only transiently impacted the microbiota, suggesting that this cycle of activity was only mildly to moderately stressful.

In Nicaragua, hunting dogs are an important part of the community, but often are left to roam, share the food of the family that owns them and are at risk for malnutrition and dehydration. In a study evaluating hair cortisol as a marker of chronic stress in 454 Nicaraguan hunting dogs (Bowland et al.), cortisol concentrations were higher in dogs with light-colored fur, and those with low body condition score (emaciated or thin). While excessive body fat can lead to progression of osteoarthritis (4), emaciation is associated with increased stress.

In addition to physiological stress, working dogs undergo physical stress from repetitive motion. This type of stress typically manifests as musculoskeletal injury, altered mechanics (joint range of motion), swelling, pain or lameness. In a longitudinal study of 323 New Zealand working farm dogs (Isaksen et al.), 57% of dogs developed at least one musculoskeletal abnormality as determined by a veterinary examination. The carpus and stifle were most commonly associated with reduced range of motion, whereas pain was most commonly found in the hip joint. Over half of the dogs that developed one abnormality, also developed a second abnormality over time. This study is one of the first to document increased risk of further injury following development of a primary musculoskeletal problem.

The anatomical structure of a working dog, in combination with the dog's kinematics (5), may impact the amount of strength and power it may generate, as well as affect its risk of development of musculoskeletal injuries. In a comprehensive review, Zink and Schlehr synthesized published data with expert observations to systematically describe the critical relationship between structure and function of the canine musculoskeletal system. Further research and understanding of the link between form and both skill and injury will be vital to improving performance and longevity of working dogs. Some of the common structural abnormalities in working dogs involve the lower back and pelvis. Even with normal structure, the repeated motion of jumping up onto raised surfaces or standing on the hind limbs to search elevated locations can put strain on the low back. Computed tomography was used to retrospectively evaluate the pelvis and lumbosacral spine in two different working breeds. The sacral iliac joint, which is the connection between the pelvis and spine, was evaluated for lesions in a retrospective study of 22 working Labrador retrievers by Carnevale et al., The methodology may be useful for future minimally invasive evaluations of working dogs with low back pain. Computed tomographic images of working military German Shepherd and Belgian Malinois lumbar vertebra were compared (Dragicevich et al.), the German Shepherd dogs had a higher incidence of both funnel-shaped lumbar vertebral foramina and articular process dysplasia malformations which may be associated with low back pain.

In addition to selecting potential working dogs based on a physical structure that will support the expected workload, preventing injury requires strategies of physical fitness to build muscle strength and flexibility. A novel approach to standardized training and canine fitness testing was described by Farr et al. Longitudinal studies will be necessary to determine the impact of fitness and conditioning on injury prevention, but standardized testing will be invaluable in such studies.

## Odor Detection

This special issue covers an array of topics relevant to the performance, capabilities, and assessment of detection dogs. The use of conservation detection dogs is growing in popularity and in success [e.g., (6–8)]. Fukuzawa and Shibata investigated dogs' detection limits for the Carolina anole and found that dogs could successfully identify samples from enclosures housing a wide variety of anole population densities. This fundamental research is critical to the continued development of detection dogs as a new tool in conservation work battling invasive species as well as acting as an important contribution to a promising and burgeoning field for the scientific use of detection canines in conservation.

In a remarkable synergy across four laboratories and international borders, the importance of standardization and attention to subtle procedural differences in detection dog training and evaluation emerged as the zeitgeist for this field. Guest et al., humbly share an important lesson learned with their medical detection canines. When a precipitous drop in performance was noted, instead of simply moving on, they investigated the cause which revealed that an easily missed difference in the processing of urine samples (whether a “dip stick” for urinalysis was placed in the sample) was aiding the dogs in the initial training. This highlights the importance of controlling every step along sample collection and processing as well as a constant evaluation of ongoing performance. The future of medical detection dog research will likely require much more attention to every detail of sample collection and processing steps to help move this field forward.

In another investigation into the effects of subtle differences in procedure on medical detection dogs' performance, Essler et al. demonstrate that the topography of the alert behavior (stop and stare vs. a sit) can have significant impacts on sampling behavior. Dogs that made a stop and stare alert showed more differentiation in sampling times between sample types (e.g., targets and non-targets) than did dogs that made a sit response. Continuing in the series of investigations on important methodological variables DeChant et al. evaluated the effect of each handler's knowledge level for the search task on team performance metrics. When handlers had more information about the number of targets to find, and whether certain areas may be “blank,” search behavior changed compared to teams without such knowledge. Teams spent more time searching and the dog more time looking back toward the handler when they had less knowledge of the search compared to teams that had more knowledge. In addition, there was no overall difference in performance when a trained researcher was monitoring searches in a single-blind (handler blind but researcher not) compared to a double-blind (handler and researcher blind) test.

The assessment of canine performance and measurement of a dog's capacity for high performance is critical for the successful procurement and training of detection dogs. In a series of studies, Rooney and Clark developed a monitoring instrument for detection dog performance in which observers rate dogs' performance during searches. Through systematic investigation, the authors investigated how subtle differences in the way the scoring system is presented and conducted (i.e., adding benchmarks to the rating scale or having handlers rate their own dogs) can lead to important differences in ratings (Clark and Rooney; Clark et al.). Together, these papers highlight the non-trivial nature of developing ratings for detection dog performance, and how minor changes in how questions are posed, whether benchmarks are given, or familiarity of the rater to the dog being rated, can all impact the results.

In addition to developing performance metrics, it is also important to identify and describe the behavioral characteristics associated with explosives detection performance. Lazarowski et al. review the prior research and identify behavioral characteristics consistently associated with optimal explosives detection dog performance. The authors focus on three broad categories, detection characteristics, overall trainability, and environmental soundness. Together, this review highlights how individual behavioral characteristics can have important functional consequences on a dog's suitability as an explosives detection dog and will lead to new and exciting research directions.

Lastly, two additional extensive narrative reviews provide thorough summaries of even more methodological considerations in canine detection work. In the first review, Lazarowski et al. discuss many of the same methodological considerations raised elsewhere in this Research Topic issue as well as many other considerations. This review will likely become a critical reference material for those interested in starting detection canine research. Similarly, Simon et al. provide a thorough review of the types of canine training aids. Training aids are the odor sources used for detection dogs. In many circumstances, the target material dogs are trained to detect (e.g., explosives) maybe too dangerous for frequent training. Therefore, the relevant odor needs to be presented in a safe and reliable manner and this extensive review highlights the varying approaches and limitations of each. As the use of detection dogs extends into new areas (biohazard detection such as COVID-19 or detection of critically endangered animals) methods to collect and store relevant target odorants will become of greater importance, and this review is a great starting point to learn about the benefits and limitations of each training aid technology.

## Human-Animal Bond

Last, but not least, it is important to recognize that nearly all working dogs work within the context of a human partnership. The human-animal bond is therefore a critical aspect to evaluate and perhaps most important within the context of service dogs. Lloyd et al. examined feelings and experiences when a service dog partnership has come to an end. Their results highlight the grief and negative feelings associated with the loss of a guide dog drawing attention to the similarity of grief following the loss of a pet dog. These results underscore the importance of considering the human animal bond in the working dog industry, especially following the loss of a service dog.

The human-animal bond also comes into play when considering the training methodology selected. Different working dog (and pet dog) domains can have different training methodologies to achieve the goals needed for the dog's assigned tasks. Many methods are employed including the use of aversive stimuli such as electronic collars. China et al. show that when comparing the efficiency and performance of dogs trained on recall and sit using an electronic collar or positive reinforcement, there was no overall difference in the number of commands disobeyed between groups. This highlights that positive reinforcement procedures can be just as efficient and effective as electronic collars. Positive reinforcement procedures may build a stronger human-animal bond and avoid the potential welfare risks from aversive techniques, therefore, identifying training methods for working dogs that are not only highly effective but also promote animal welfare and the human-animal bond is an important future direction for this field. Together, these two important papers highlight the need for additional research investigating the human-animal bond in the context of working dogs, which still receives little research attention.

## Overall Summary

In order to maintain form and function, awareness of the physical and behavioral stressors of working dogs is necessary. These studies have shown that although working dogs are resilient, they are often at risk of stressors that can impact their welfare and performance. The topics of working dog welfare, nutrition, hydration, physical fitness and exercise are all timely topics that warrant continued investigation.

## Author Contributions

All authors listed have made a substantial, direct and intellectual contribution to the work, and approved it for publication.

## Conflict of Interest

The authors declare that the research was conducted in the absence of any commercial or financial relationships that could be construed as a potential conflict of interest.

## Publisher's Note

All claims expressed in this article are solely those of the authors and do not necessarily represent those of their affiliated organizations, or those of the publisher, the editors and the reviewers. Any product that may be evaluated in this article, or claim that may be made by its manufacturer, is not guaranteed or endorsed by the publisher.
